# Ethical Aspects of Artificial Intelligence in Precision Medicine for Obesity Management

**DOI:** 10.31662/jmaj.2025-0412

**Published:** 2026-05-08

**Authors:** Marianela Bastías-Pérez

**Affiliations:** 1 Núcleo de Investigación en Nutrición y Ciencias Alimentarias (NINCAL), Facultad de Salud y Ciencias Sociales, Universidad de Las Américas, Santiago, Chile; 2 Centro de Investigación en Ciencias Biológicas y Químicas (CICBQ), Facultad de Medicina Veterinaria y Agronomía, Universidad de las Américas, Santiago, Chile; 3 Centro de Investigación Biomédica en Red (CIBER) de Fisiopatología de la Obesidad y Nutrición (CIBEROBN), Instituto de Salud Carlos III (ISCIII), Madrid, Spain

**Keywords:** obesity, artificial intelligence, precision medicine, ethics, equity, privacy

## Abstract

Artificial intelligence (AI) has established itself as a key tool in the development of precision medicine, offering opportunities to improve risk prediction, early diagnosis, and personalized treatments for obesity. However, its implementation poses ethical challenges that cannot be ignored. The use of sensitive data such as genetic, metabolic, and behavioral information raises questions about privacy, security, and informed consent. Furthermore, the existence of algorithmic biases can reproduce inequities and limit the validity of models in diverse populations, particularly in low-resource settings.

Another critical aspect is the lack of transparency in many AI systems, whose operation as “black boxes” makes it difficult to interpret their recommendations and weakens trust between professionals and patients. Legal accountability for errors in clinical decision-making, limited accessibility of high-cost technologies, and the imperative to respect patient autonomy and cultural context highlight the urgency of establishing a clear and enforceable ethical framework.

This narrative review analyzes the main ethical dilemmas associated with the use of AI in precision medicine for obesity, highlighting challenges related to equity, transparency, social justice, and clinical accountability. The need for responsible technological development is raised, ensuring respect for patients’ rights and promoting equitable use of these innovative tools in addressing one of the most complex and prevalent diseases worldwide.

## Introduction

Obesity is currently one of the main global public health problems, with a prevalence that has steadily increased in recent decades and affects more than one billion people worldwide, including children and adolescents, according to recent estimates by the World Health Organization ^(1)^. This multifactorial and heterogeneous condition is closely associated with comorbidities such as type 2 diabetes mellitus, high blood pressure, dyslipidemia, and cardiovascular disease, which significantly increase the global burden of disease and healthcare costs ^(2)^.

In this context, precision medicine has emerged as an innovative paradigm that seeks to adapt therapeutic interventions to the biological, genetic, and environmental profile of each individual, overcoming the limitations of generalized approaches ^(3)^. The integration of omics tools and clinical and behavioral biomarkers allows for the identification of risk profiles and the design of more effective and sustainable long-term strategies ^(4)^.

At the same time, the development of artificial intelligence (AI) has revolutionized the analysis of large volumes of health data, offering the possibility of detecting complex patterns and generating predictive models applicable to the management of metabolic diseases and obesity in particular. Recent evidence has shown that AI can support risk stratification, diet personalization, prediction of response to pharmacotherapy, and real-time monitoring using digital devices ^(5), (6)^. However, along with these opportunities, ethical challenges emerge that require special attention. The management of sensitive data, algorithmic biases, model transparency, and equitable access are central aspects that must be addressed to ensure the responsible use of these technologies ^(7), (8)^. Therefore, this narrative review aims to analyze the main ethical dilemmas associated with the use of AI in precision medicine applied to obesity, highlighting its clinical, social, and regulatory implications. Because this work follows a narrative review approach, the literature included was selected based on its conceptual relevance rather than predefined systematic criteria, and the seven ethical domains discussed in this article emerged from recurrent themes identified across the reviewed publications. To enhance transparency, peer-reviewed articles were identified through non-systematic searches in major biomedical databases (PubMed, Scopus, and Web of Science), prioritizing publications from the last decade and including landmark works when relevant. Inclusion was guided by thematic relevance to AI ethics and obesity, while non-peer-reviewed or out-of-scope sources were excluded. The ethical domains analyzed were therefore derived inductively from converging themes across the selected literature.

## Ethical Aspects of Using AI in Precision Medicine for Obesity

**Figure 1.**
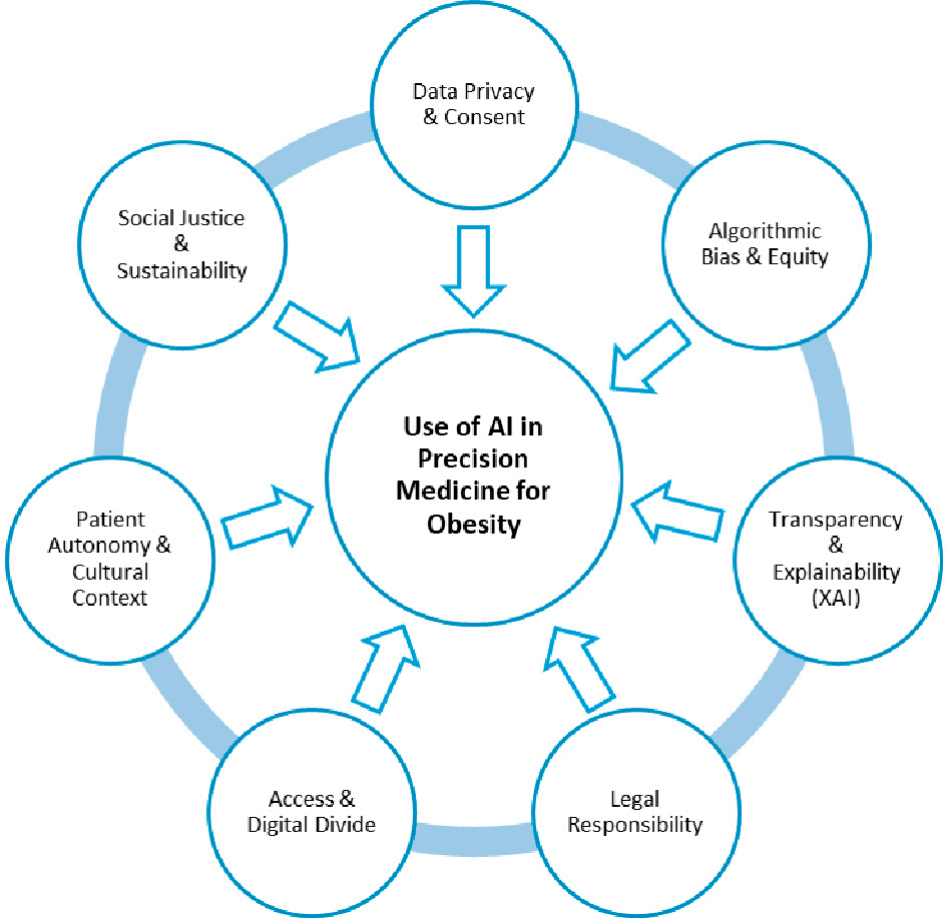
Key ethical aspects of the use of AI in precision medicine applied to obesity. The diagram shows seven critical dimensions: data privacy and consent, algorithmic bias and fairness, transparency and explainability, legal accountability, access and technology gap, autonomy and cultural context, and social justice and sustainability. These areas represent the key ethical dilemmas to be addressed for a responsible and equitable implementation of AI in this field. AI: artificial intelligence.

While AI offers important opportunities for personalization and decision support, its adoption in healthcare should not be assumed to be inherently beneficial or inevitable. Structural risks must also be considered, including the potential erosion of privacy due to continuous data collection, the algorithmization of lifestyle behaviors through automated recommendations, and forms of implicit or non-transparent nudging that may influence patient choices without explicit consent. In addition, AI systems can inadvertently reinforce existing inequities if trained on unrepresentative data or implemented unevenly across populations. Acknowledging these structural risks is essential to ensure that AI contributes to obesity care without undermining autonomy, fairness, or trust. Figure 1 illustrates the main ethical domains associated with the use of artificial intelligence in precision medicine for obesity.

### Privacy and data protection

The implementation of AI in precision medicine for obesity relies heavily on the collection, storage, and analysis of highly sensitive information. This data include genomic information, the gut microbiome, metabolic biomarkers, electronic medical records, and data generated in real time by wearables and mobile applications. The wealth of this information offers great potential for improving the prevention and treatment of obesity, but also opens the door to significant privacy and security risks ^(9)^.

One of the main dilemmas is informed consent. In many cases, patients are unaware of the scope of their data use, as well as the possibility that it may be used for secondary purposes, such as commercial research, targeted marketing, or health insurance development. This scenario creates a tension between the need to share data for scientific progress and patients’ right to govern the use of their personal information ^(10)^.

Another critical aspect is vulnerability to cyberattacks. Health databases, particularly those containing genetic information, are a frequent target for unauthorized access due to their high economic and scientific value ^(11)^. Security breaches in these systems can lead to consequences of varying magnitude, including the misuse of personal data in employment or health insurance contexts, as well as the possibility of social stigmatization. At the collective level, the exposure of sensitive information can undermine trust in digital health platforms and limit patients’ willingness to share data necessary for the development of precision medicine ^(12)^.

Furthermore, health data on obesity often comes from multiple and heterogeneous sources (hospitals, mobile applications, wearable devices), which poses interoperability issues and increases the likelihood of anonymization errors. Even when data are anonymized, recent studies have shown that re-identification techniques can reconstruct individual profiles based on patterns of supposedly protected information ^(12)^.

From an ethical perspective, the central challenge lies in establishing robust and universal regulatory frameworks that guarantee data protection at all stages of its lifecycle: collection, storage, analysis, and sharing. International organizations such as the European Union, with the General Data Protection Regulation, have made progress in this regard, but significant regulatory gaps remain in low- and middle-income regions ^(13)^. In Latin America, where obesity rates are rising and healthcare systems still face structural limitations, implementing AI without adequate security measures could exacerbate inequalities rather than mitigate them ^(14)^.

In this scenario, patient trust becomes a fundamental element. If individuals perceive that their data could be misused or insecure, resistance to sharing information is likely, which would limit the development of AI in precision medicine. Therefore, solutions must combine advanced cybersecurity technology, enhanced anonymization, clear informed consent protocols, and, above all, transparency in data management so that patients understand how and for what purposes their information will be used ^(15)^.

In short, privacy and data protection in the use of AI applied to obesity is not only a technical challenge, but also an ethical dilemma that involves patients’ trust, autonomy, and fundamental rights. Resolving these challenges is essential for these technologies to be legitimately and equitably consolidated in healthcare systems.

Beyond clinical environments, AI systems used in direct-to-consumer tools (such as smartphone applications, wearable devices, and commercial lifestyle platforms) raise additional ethical challenges. These technologies often rely on continuous data collection, unclear accountability structures, and persuasive design mechanisms that may nudge users toward specific behaviors without explicit awareness or consent. At the population level, AI-driven monitoring tools can also be incorporated into public-health governance, potentially shaping behavioral policies in ways that risk surveillance, discrimination, or disproportionate pressure on individuals with obesity. Addressing these consumer-level and public-health dimensions is essential to ensure that AI-supported obesity interventions remain transparent, equitable, and respectful of individual autonomy.

### Algorithmic biases and health equity

One of the main ethical risks in the application of AI in precision medicine for obesity is the presence of algorithmic biases, which can compromise the validity of predictions and reinforce existing health inequities. These biases largely stem from the quality and representativeness of the data used to train the models.

Most AI algorithms in health have been developed using data from specific populations, primarily from Europe and North America, which creates limitations for their applicability in regions with distinct genetic, cultural, and socioeconomic diversity, such as Latin America, Africa, or Asia ^(16)^. Consequently, a model trained with information from a homogeneous population may underestimate the risk in certain groups or overestimate it in others, resulting in less accurate diagnoses and inappropriate therapeutic recommendations.

Biases are not limited to input data but can accumulate throughout the entire AI lifecycle, from variable selection to result interpretation. For example, if clinical databases systematically exclude ethnic minorities or low-income individuals, algorithms will reproduce this invisibility, perpetuating the exclusion of already vulnerable groups ^(17)^.

In obesity, where multiple social determinants play a role, such as unequal access to healthy foods, education, income, and opportunities for physical activity, the failure to integrate these variables into models can lead to reductionist interpretations, attributing risk solely to biological or genetic factors and downplaying the importance of social determinants of health ^(18)^.

The ethical impact of this problem is significant, as biased AI not only limits its clinical utility but also exacerbates structural inequities. Recent evidence has shown that algorithms trained with data from privileged populations tend to generate more effective recommendations for these groups, while offering less accurate or even erroneous solutions in marginalized communities, contributing to the widening of the healthcare technology gap ^(19), (20)^. To address this challenge, several authors have proposed the need to increase the diversity of training sets by incorporating populations from different ethnicities, socioeconomic backgrounds, and geographic regions. Furthermore, the implementation of algorithmic bias audits to identify and correct flaws in models before clinical application is recommended, as well as the development of regulatory frameworks that require AI providers to report the composition of their databases and the identified biases ^(21)^.

Ultimately, the presence of algorithmic bias in AI applied to obesity is not only a technical problem, but also an ethical and social dilemma that affects trust in these technologies and limits their translational potential. Overcoming this challenge requires an interdisciplinary engagement involving developers, clinicians, bioethicists, and policymakers to promote more just, equitable, and patient-centered AI.

### Transparency and explainability of algorithms

One of the dilemmas to consider when applying AI to precision medicine for obesity is the lack of transparency in the models used. Many current algorithms, especially those based on deep learning, operate as “black boxes”: they generate predictions or recommendations without users (physicians or patients) clearly understanding how these conclusions were reached ^(19)^.

This opacity raises several ethical issues. First, it affects physician-patient trust. The therapeutic relationship is based on the practitioner’s ability to explain and justify their decisions, which is limited if the recommendations come from a system whose internal workings are not comprehensible ^(20)^. Second, it impedes accountability; if an algorithm suggests a treatment that is inappropriate or risky, how can the physician justify that choice if they do not understand the criteria used by the model?

To address the lack of transparency in many artificial intelligence systems, the concept of explainable AI has been developed in recent years. Its purpose is to increase the interpretability, auditability, and understandability of models for both healthcare professionals and patients ^(21)^. This approach seeks to clearly identify which variables contribute most to risk prediction, how different factors are weighted, such as genetic versus behavioral determinants, and what data support the recommendation of a specific intervention. In this way, explainable AI aims to facilitate trust in algorithmic systems and support informed clinical decision-making in obesity management ^(21)^.

The lack of transparency in artificial intelligence systems also has regulatory implications. In response to this situation, various international agencies and digital governance frameworks have begun to establish guidelines requiring AI developers in healthcare to report minimum information on model design, the origin of the data used, and potential associated biases ^(22)^. The absence of this type of oversight could favor the incorporation of opaque systems into clinical practice, with consequences for patient safety and the definition of professional responsibilities.

Transparency is also linked to the possibility of more informed patient participation. In obesity management, where interventions often involve changes in dietary and behavioral habits, it is necessary for individuals to understand the rationale behind the recommendations generated by algorithms. This helps respect their autonomy and can promote treatment adherence.

However, the interpretability of systems is not always straightforward. There is an inherent tension between the accuracy of more complex models and the ability to explain their results, given that algorithms with greater predictive power are often less transparent, while simpler models offer greater interpretability at the cost of lower accuracy ^(23)^. This balance is recognized as one of the main ethical and methodological challenges in the development and implementation of AI applications in healthcare ^(23)^. Overall, moving toward explainable and auditable AI systems appears to be a fundamental requirement for promoting their safe, ethical, and effective integration into precision medicine aimed at managing obesity.

Table 1 summarizes the main ethical challenges, associated risks, and potential solutions related to the use of artificial intelligence in precision medicine for obesity.

**Table 1.** Ethical Challenges of Artificial Intelligence in Precision Medicine for Obesity.

Ethical Issue	Main Risks	Potential Solutions	References
Data privacy and consent	Unauthorized use or breaches of genomic and clinical data	Robust informed consent, advanced anonymization, and international data protection frameworks	(9-15)
Algorithmic bias and equity	Non-representative datasets; inaccurate predictions for vulnerable groups	Inclusion of diverse populations, regular bias audits, and transparent reporting of training data	(16-21)
Transparency and explainability	“Black box” algorithms; limited trust and accountability	Development of Explainable AI (XAI), external audits, and clear methodological documentation	(19-23)
Legal responsibility	Unclear accountability in adverse outcomes	Shared responsibility protocols, regulatory standards, AI as support, not replacement, of clinical judgment	(24-27)
Access and digital divide	High cost of genomics, big data, and wearables; unequal access to technology	Public subsidies, low-cost adapted tools, international collaboration, and equitable policy design	(28-30)
Patient autonomy and cultural context	Recommendations misaligned with values, culture, or economic context	Cultural adaptation of AI tools, shared decision-making, and respect for patient preferences	(31-35)
Social justice and sustainability	Concentration of benefits in high-income settings; corporate dependency	Open-source platforms, public investment, global cooperation, and environmentally sustainable AI models	(36-40)

AI: artificial intelligence.

## Legal Liability and Clinical Decision-Making

The integration of AI in precision medicine applied to obesity raises a relevant debate around legal liability. In scenarios where an algorithm suggests an intervention that proves ineffective or even harmful, there is a need to clearly define which actors should assume responsibility: the medical team that interprets the results, the institution that implements the system, the developers who design the software, or the regulatory bodies that authorize its use ^(24)^.

Currently, most AI systems in healthcare are considered clinical decision support tools and not substitutes for professional judgment. From this perspective, ultimate responsibility rests with healthcare professionals, who must critically interpret recommendations before applying them to the patient ^(25)^. However, as algorithms become more autonomous and sophisticated, the line between support and decision-making tends to become blurred, increasing the need for specific regulatory frameworks that delineate responsibilities.

The complexity of this dilemma can be illustrated in the context of prescribing personalized AI-based diets or pharmacological treatments. If a patient experiences adverse effects resulting from an automated recommendation, it is pertinent to question whether the practitioner acted with due diligence in relying on the tool and, at the same time, whether the developers fulfilled their obligation to adequately report the limitations and potential biases of their models ^(26)^. This problem is even more relevant in obesity, where the interaction of genetic, metabolic, behavioral, and social factors makes it difficult for algorithms to capture the full complexity of the phenomenon, which can lead to incomplete or inappropriate recommendations with additional clinical and legal consequences ^(27)^.

To address these challenges, we propose moving toward shared responsibility protocols that precisely define the role of each actor. This requires healthcare professionals to maintain clinical judgment as a central axis in decision-making, and software developers to ensure the transparency and validation of models. While transparency is essential for shared responsibility frameworks, it does not require full disclosure of proprietary source code. Instead, transparency can be achieved through independent audits, performance documentation, and regulatory oversight, which allow model validation while safeguarding intellectual property rights. Health care institutions should oversee the responsible implementation of these technologies, and regulatory bodies should establish clear frameworks regarding safety, validation, and legal liability. The lack of a clear definition of these aspects can generate legal uncertainty for both patients and professionals, affecting confidence in the implementation of AI in clinical practice. In this sense, the discussion about legal liability is not only a technical or legal issue, but also a fundamental ethical requirement to ensure safety, trust, and legitimacy in the use of AI in obesity.

### Access and technology gap

One of the main ethical challenges in the implementation of AI and precision medicine in obesity is the inequality in access to these technologies. Many recent advances, such as genomic sequencing, microbiome analysis, the use of big data systems, or advanced wearable devices, require expensive infrastructure and highly specialized human resources. This has limited their availability primarily to high-income countries or cutting-edge research centers, while a large part of the world’s population continues to face economic and technological barriers that restrict the benefits of these innovations ^(28)^.

In low- and middle-income countries, technology gaps are expressed at several levels. Digital infrastructure is often limited, with incomplete or nonexistent clinical records; the availability of professionals trained in bioinformatics, artificial intelligence, and data management is limited; and access to digital health devices, such as continuous glucose monitors, activity sensors, or mobile tracking applications, remains restricted. These conditions have contributed to what has been called the “reverse digital dividend,” a phenomenon in which, rather than reducing health inequities, the incorporation of new technologies tends to widen them by primarily benefiting those who already have resources and access to advanced services, while the most vulnerable populations are left behind ^(29)^.

In the case of obesity, this situation is especially relevant, given that the condition disproportionately affects low-income communities, where the availability of healthy foods is often limited and conditions for physical activity are less favorable. The application of algorithms trained with data from privileged populations in highly vulnerable contexts can lead to irrelevant or difficult-to-implement recommendations, with the risk of perpetuating structural inequities ^(30)^.

From an ethical perspective, health equity implies that technological innovation must be designed and implemented considering the diversity of populations, and not only the needs of those with greater purchasing power or access to highly developed health systems. In this regard, evidence has highlighted the importance of promoting the development of low-cost tools adapted to contexts with limited resources, strengthening collaboration to share infrastructure, databases, and technical knowledge, and promoting public funding programs that facilitate access to digital health technologies. Likewise, it is essential to ensure the inclusion of diverse populations in studies and clinical trials that validate the use of AI in obesity. Bridging the technological gap is not only a matter of social justice, but also a requirement for scientific efficacy. Without population diversity, AI models will remain limited, and their predictions will lack universal validity. Therefore, ensuring equitable access to AI and precision medicine is both an ethical and practical imperative to achieve a real impact on global obesity management.

### Patient autonomy and cultural context

Precision medicine and AI offer great potential for personalizing obesity treatment. However, these technologies must be implemented with respect for patient autonomy and cultural context. Otherwise, they run the risk of imposing recommendations that are unrealistic, culturally inappropriate, or that reduce individual decision-making capacity ^(31)^.

A fundamental ethical aspect is respect for autonomy. AI can generate highly accurate predictions about which diet or drug treatment would be most effective, but these suggestions should be presented as supportive tools rather than rigid prescriptions. Patients must retain the ability to accept, adapt, or reject the recommendations, in accordance with their values, beliefs, and personal circumstances ^(32)^. Furthermore, algorithms trained on specific populations can generate nutritional or behavioral plans disconnected from the cultural reality of certain groups. For example, a system that recommends foods that are beyond the reach of the patient or that are not part of a community’s regular diet can lead to rejection and poor adherence. In obesity, where changing habits is key, cultural sensitivity is essential to achieving sustainable changes ^(33)^.

Another key point is clear communication between healthcare professionals and patients. If an algorithm suggests an intervention, the physician must be able to explain it in understandable terms, facilitating shared decision-making. This strengthens trust and adherence and prevents AI from being perceived as a dehumanizing substitute for clinical practice ^(34)^. Finally, respect for patient autonomy remains a central ethical obligation grounded in principlist ethics, relational autonomy, and procedural justice. Within this framework, attention to cultural diversity should be understood as a necessary condition for the meaningful realization of these principles in practice, rather than as an independent foundational norm. Cultural context shapes communication, trust, and the interpretation of health recommendations, thereby influencing how autonomy and justice are effectively exercised in clinical encounters. In this sense, diversity is not only ethically relevant but also a determinant of clinical effectiveness, as interventions that acknowledge patient values and sociocultural conditions tend to achieve higher adherence and sustainability. Evidence shows that interventions that ignore cultural context have significantly lower adherence rates, while those that integrate patient values and traditions achieve more sustainable results ^(35)^. Thus, AI applied to obesity should be conceived as a flexible ally, capable of adapting to cultural diversity and reinforcing, rather than replacing, patient autonomy in their care process.

### Social justice and sustainability

The implementation of AI in precision medicine for obesity poses not only technical and clinical challenges but also significant social implications. These technologies, if developed and applied primarily in high-income countries or private institutions, may contribute to reinforcing health inequalities rather than reducing them ^(36)^. Obesity disproportionately affects low-income communities, where factors such as food insecurity, limited availability of healthy foods, lack of safe spaces for physical activity, and precarious access to health services increase vulnerability. In this context, it is problematic that many AI systems for obesity are designed and validated in privileged settings without adequately considering the reality of the most affected populations ^(37)^.

From a social perspective, the challenge is not only to perfect the technology but to ensure that its implementation effectively contributes to reducing health inequalities. In this sense, it is essential to promote the inclusion of diverse populations in databases, train algorithms in representative contexts, and establish public policies aimed at democratizing access to these innovations ^(38)^.

Sustainability is another key aspect. The development and maintenance of AI systems in health require large volumes of data, energy, and technological resources, which raises questions about their long-term viability, particularly in countries with fragile health systems ^(39)^. Furthermore, the dependence on private corporations for the design and control of these technologies can generate structural dependency and limit the autonomy of public health systems. Given this scenario, evidence suggests the importance of promoting open-source models and collaborative platforms that allow for the equitable sharing of algorithms and data, boosting public investment in applied research on obesity, establishing international collaboration frameworks that strengthen capacities in the most vulnerable regions, and incorporating principles of environmental and economic sustainability into the design of digital health technologies ^(40)^.

Together, social justice and sustainability must be considered central elements in the integration of AI into precision medicine applied to obesity. Only through this approach will it be possible to ensure that these tools contribute to strengthening more equitable, inclusive, and resilient health systems.

## Conclusions and Future Perspectives

Artificial intelligence applied to precision medicine represents an opportunity to transform the approach to obesity in the areas of prevention, diagnosis, and treatment. Its ability to integrate genomic, metabolic, behavioral, and environmental data allows for the design of more targeted and personalized interventions, which could overcome some of the limitations of conventional approaches. However, this potential must be analyzed considering the ethical dilemmas that accompany its implementation. Data privacy and protection are recognized as a priority, given the sensitivity of the information involved and the possibility of inappropriate use. Algorithmic biases also represent a challenge, as they compromise the validity of models and can amplify inequities in vulnerable populations. Similarly, the transparency and explainability of systems are essential to maintain trust in the doctor-patient relationship and ensure accountability.

The definition of legal responsibilities is another critical point, as a lack of clarity in this area can affect both patient safety and the legal security of healthcare professionals. Added to this are the problems arising from technological gaps, which threaten to concentrate the benefits of these innovations on those who already have greater resources, to the detriment of the most needy populations. Furthermore, respect for patient autonomy and consideration of the cultural context are essential elements for the acceptability and sustainability of interventions. Finally, social justice and sustainability must be guiding principles so that the implementation of these technologies contributes to reducing, rather than perpetuating, health inequalities.

Looking ahead, the literature emphasizes the need for robust regulatory frameworks that establish standards for safety, transparency, and accountability; the diversification of data sets to improve the representativeness of algorithms; and the promotion of explainable AI approaches that balance predictive accuracy with interpretability. The importance of promoting public policies that facilitate equitable access and reduce the digital divide is also highlighted, as well as investing in ethical and digital education for both healthcare professionals and patients. Overall, AI has the potential to become a strategic resource in addressing obesity, provided its development and application are guided by principles of justice, equity, autonomy, and respect for human rights. The consolidation of this approach will depend on the ability to integrate these technologies in a responsible, sustainable, and human-centered manner, ensuring that the benefits are distributed fairly throughout society.

### Limitations

As a narrative review, the scope of this work reflects the conceptual focus of the selected literature. The ethical domains presented here synthesize the major themes currently highlighted in discussions on AI and precision medicine. Future scholarship may expand these reflections by incorporating empirical data and perspectives from diverse clinical and regulatory contexts.

## Article Information

### Conflicts of Interest

None

### Acknowledgments

The author acknowledges the support of the project *“Impact of intermittent fasting on brown adipose tissue activation and batokine production in obese mouse models”* (PIR2025_06), Universidad de Las Américas, Chile, as well as funding from *Fondo Mujeres UDLA+i* (INID240002), Universidad de Las Américas, Chile.

### Author Contributions

Manuscript preparation: Marianela Bastías-Pérez.

### IRB Approval Code and Name of the Institution

Not applicable, as this study is a review.
